# Neural responses to apparent motion can be predicted by responses to non-moving stimuli

**DOI:** 10.1016/j.neuroimage.2020.116973

**Published:** 2020-09

**Authors:** Marlene Poncet, Justin M. Ales

**Affiliations:** School of Psychology, University of St Andrews, UK

**Keywords:** Apparent motion, EEG, Long-range, Short-range, Spatio-temporal interactions

## Abstract

When two objects are presented in alternation at two locations, they are seen as a single object moving from one location to the other. This apparent motion (AM) percept is experienced for objects located at short and also at long distances. However, current models cannot explain how the brain integrates information over large distances to create such long-range AM. This study investigates the neural markers of AM by parcelling out the contribution of spatial and temporal interactions not specific to motion. In two experiments, participants’ EEG was recorded while they viewed two stimuli inducing AM. Different combinations of these stimuli were also shown in a static context to predict an AM neural response where no motion is perceived. We compared the goodness of fit between these different predictions and found consistent results in both experiments. At short-range, the addition of the inhibitory spatial and temporal interactions not specific to motion improved the AM prediction. However, there was no indication that spatial or temporal non-linear interactions were present at long-range. This suggests that short- and long-range AM rely on different neural mechanisms. Importantly, our results also show that at both short- and long-range, responses generated by a moving stimulus could be well predicted from conditions in which no motion is perceived. That is, the EEG response to a moving stimulus is simply a combination of individual responses to non-moving stimuli. This demonstrates a dissociation between the brain response and the subjective percept of motion.

## Highlights

• EEG responses are inhibited by spatial and temporal stimulus interactions.
• These interactions are important for motion at short but not at long distances.
• We find no trace of a specific neural signature of motion perception.
• Neural responses to motion are well predicted by responses to non-moving stimuli.

## 1 Introduction

Apparent motion (AM) is the perception of an object moving from one location to another location distant in space and/or in time (Sekuler, 1996). This phenomenon is evoked only for a specific range of temporal parameters. For example, if the two objects are presented very fast (over 14 ​Hz), participants report seeing two independent flickering objects (Ekroll et al., 2008; Kolers, 1972). On the other hand, AM is largely immune to the identity of the objects (Chong et al., 2014; Hidaka et al., 2011; Kolers and Pomerantz, 1971; Nishida et al., 2007; Tse and Caplovitz, 2006) and can be perceived for two objects separated by short or long spatial distances, even though different mechanisms might underlie these short and long-range motion systems (Braddick, 1974; Kolers, 1972; Larsen et al., 1983; Zhuo et al., 2003; but see Cavanagh and Mather, 1989). The neural mechanisms of motion perception over short distances are well understood, but they remain largely unknown for objects separated by large distances. The aim of this study is to a) uncover the neural mechanisms underlying the percept of AM by controlling for temporal and spatial factors not specific to motion perception and b) determine the differences and similarities in these neural mechanisms for stimuli separated by short and long spatial distances.

Current results and models of motion can account for AM at short-range but not at long-range. By short-range, we mean stimuli that are presented within approximately the size of V1 receptive fields. In this case, direction selective neurons in V1 respond to the presentation of short-range AM similarly as they would respond for real motion. However, when stimuli are long-range, that is separated by distances larger than V1 receptive fields, the responses of direction selective V1 neurons dramatically decrease even though motion is still perceived (Churchland et al., 2005; Livingstone et al., 2001). The same limitation arises in current computational models of motion processing. They explain the perception of short-range motion very well but not the perception of long-range motion (Adelson and Bergen, 1985; Rust et al., 2006; Simoncelli and Heeger, 1998). Indeed, these models are based on V1 tuning properties and since these properties have a limited span across space and time, computational models cannot account for motion perception over large spatial distances.

Contrary to V1 cells, MT neurons have larger receptive fields and MT area was thus thought to be a good candidate for processing long-range AM (Mikami et al., 1986). However, spatial and temporal limits of direction selectivity are the same for V1 and MT neurons (Bair and Movshon, 2004; A. K. Churchland et al., 2007; M. M. Churchland et al., 2005; Livingstone et al., 2001; Pack et al., 2006). That is, MT neurons, just as V1 neurons, show motion direction selectivity for short-range AM but not for long-range AM. Importantly, MT neurons do not always correlate with motion perception (Ilg and Churan, 2004). When faced with a stimulus containing both local and global motion, human participants report perceiving the direction of the global motion whereas MT neurons recorded in macaque monkeys under the same conditions respond to the direction of the local motion (Hedges et al., 2011). Similarly, using plaids, Majaj et al. (2007) were able to show that MT neurons integrate local motion signals rather than motion signals pooled across their entire receptive field. Thus, the perceived motion direction for global motion seems to be computed in another area than MT. This suggests that the neural mechanisms supporting long-range motion are different from the ones supporting short-range motion.

On the other hand, the distinction between local and global motion might be different for AM stimuli. In AM, the motion percept relies strongly on the temporal sequence of the stimuli and there is evidence that temporal synchrony can induce non-linear neural interactions at long-range distances (Caplovitz et al., 2008). Perceptually, two objects presented successively at distinct location creates the illusion of a motion path between these locations and participants detect targets on the AM path less often than targets presented at other locations in the visual field (Yantis and Nakama, 1998). It has been proposed that this impairment, called AM masking, is due to the mental representation of the moving object on the AM path. This representation would inhibit the processing of another object on the illusory path (Hidaka et al., 2011; Hogendoorn et al., 2008; Yantis and Nakama, 1998). This is supported by fMRI studies that have reported brain activity in V1 along the AM illusory path, similar to the activity observed for a real moving object (Akselrod et al., 2015; Chong et al., 2016; Larsen et al., 2006; Muckli et al., 2005; Sterzer et al., 2006). This activation has been hypothesized to be the consequence of feedback from temporal areas (Ferrera et al., 1994; Zhuo et al., 2003) but other findings suggest that it might be the result of feedback from MT (Matsuyoshi et al., 2007; Pascual-Leone and Walsh, 2001; Silvanto et al., 2005; Vetter et al., 2015). Thus, AM seems to activate, at a population level, the representation of a moving object in V1, which inhibits the processing of other objects presented along the AM path. AM could therefore rely on neural mechanisms similar to the ones involved in real motion thanks to feedback processes. However, these findings are contrasted by other studies which did not find an activation of early visual areas on the AM path (Erlikhman and Caplovitz, 2017; T. Liu et al., 2004; Muckli et al., 2002).

An opposing hypothesis originating from the predictive-coding framework suggests that the illusory motion percept could inhibit V1 responses. Schwiedrzik et al. (2007) have found that, in addition to a general masking of targets presented along the AM path, targets that appeared in time with the AM percept were detected more often than those that appeared at an unexpected position or point in time. A following fMRI study further showed that a predictable, and thus more detectable, stimulus induces a smaller fMRI response in V1 compared to an unpredictable stimulus (Alink et al., 2010). The authors propose that, as predictive-coding models would suggest (Rao and Ballard, 1999), AM masking could be due to the inhibition of V1 as a consequence of the prediction generated by the brain for AM stimuli. Consistent with this hypothesis, Van Humbeeck et al. (2016) have shown using a V1-like population code model that the activation of V1 is too small to be perceptually relevant and instead, their model predicts a strong suppression of early sensory responses during AM. Thus, AM might not result in an activation but instead in an inhibition of V1 responses on the illusory path.

At both single-neuron and large fMRI neural population levels, no studies have yet found the source of the sensitivity to long-range AM. Here, we approached this question using EEG recordings. Earlier studies using EEG have tried to determine the source of motion processing in AM by taking the difference between the EEG response during AM and the sum (linear prediction) of the EEG response evoked for the two stimuli when they are not in an AM context (Norcia et al., 2017; Wibral et al., 2009). The reasoning is that the difference between the AM brain response and the linear prediction of two flashes where no motion is perceived should contain the specific EEG response to motion. Wibral et al. (2009) found that this difference started around 90 ​ms and lasted up to 200 ​ms after the presentation of the second stimulus. Their results also suggest that the specific response to motion originates from MT and they therefore propose that, in light with previous results, AM is the consequence of MT feedback to early visual areas. In a different paradigm, using steady state visual evoked potential (SSVEP), Norcia et al. (2017) found that the EEG responses to AM were around two times smaller than a linear prediction, suggesting a strong inhibition of responses during AM. Note that Wibral et al. (2009) did not find such inhibition effect in their results. Thus, both an activation from MT or an inhibition of neural responses have been found in EEG studies. In addition, these studies did not control for temporal interactions that are not related to motion processes. Here, we used a similar methodological approach but we systematically tested the contribution of the spatial and temporal components present in a motion sequence that are not specific to motion perception.

To isolate the mechanisms selective for motion using EEG activity, a comparison with a linear summation of two stimuli presented independently is not sufficient. Motion processing supposes the selectivity for a spatio-temporal sequence of a stimulus. However, pure temporal or pure spatial components, that do not produce the perception of motion, should be controlled for. That is, the EEG activity during AM can be decomposed into the following: 1) a linear prediction from the two flashes taken separately, 2) a spatial and 3) a temporal interaction that do not evoke a motion perception, and 4) a specific spatio-temporal interaction which is the source of the motion perception. In this study, we will use a protocol that allows us to determine the possible contribution of spatial and temporal components that do not involve a moving stimulus. These non-related motion interactions can then be controlled for to predict an EEG response to AM that can be compared to the brain response observed during AM. The remaining difference will be the EEG response to the spatio-temporal sequence specific to the perception of motion. Importantly, because we analyse deviations from predictions, this procedure also allows to directly compare short and long-range motion. This study should thus uncover the specific neural mechanisms at the origin of motion and the difference, if any, between short and long-range AM.

## 2 Methods

### 2.1 Participants

13 volunteers (mean age 24, 4 males) participated in Experiment 1, 17 (mean age 24, 6 males) participated in Experiment 2. Three additional volunteers participated in the experiments but were rejected from further analysis, one due to abnormal EEG, one did not complete the experiment and one due to a poor EEG signal (more than 50% of epochs were deleted because of noise artefacts). All participants were reimbursed £10 for their participation. They reported normal or corrected to normal vision and provided written informed consent. All experiments received the approval of the ethical committee of the School of Psychology, University of St Andrews.

### 2.2 Material and design

Stimuli were displayed on a gamma-corrected CRT monitor (1280x1024 pixels, 37.5 ​cm width, refresh rate 85 ​Hz) using a Linux machine with Matlab and the Psychophysics Toolbox extensions (Brainard, 1997; Kleiner et al., 2007). The stimulus presentation was controlled with a Bit# stimulus processor (Cambridge Research Systems) and synchronised with the refresh rate of the monitor and the EEG recording.

**Fig. 1:**
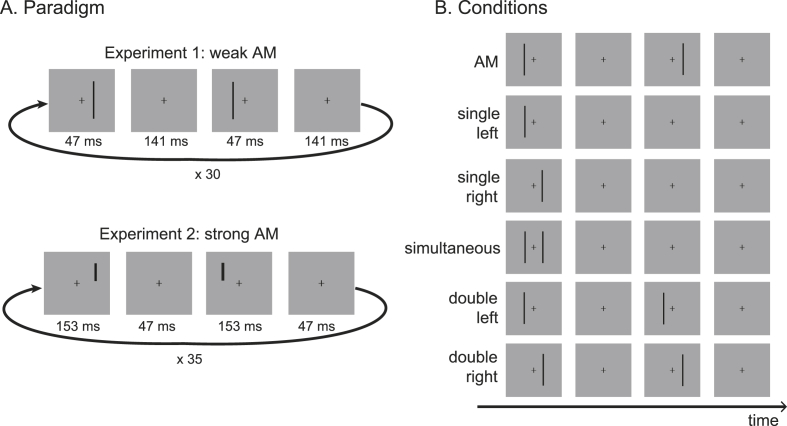
Illustration of one AM cycle (A) and the six conditions (B) used in the two experiments (scale is approximative). The stimuli were presented either at small eccentricity (short-range condition, 0.3° and 0.6° from fixation in Experiment 1 and 2 respectively) or at large eccentricity (long-range condition, 3° and 6° from fixation in Experiment 1 and 2 respectively), for a total of 12 conditions in each experiment.

In Experiment 1, the stimulus was a black vertical bar (size 0.5° ​× ​20° visual angle). A motion percept was induced by presenting the bar alternatively at two locations on the left and the right of the fixation cross at an eccentricity of 0.3° visual angle for short-range motion and 3° visual angle for long-range motion (the spatial separation between the edges of the left and right stimulus was thus 0.1° and 5.5° for the short and long-range condition respectively). The stimulus was presented for 4 screen frames (47 ​ms) in the left visual field, disappeared for 12 screen frames (141 ​ms), re-appeared in the right visual field for 4 screen frames and disappeared for 12 screen frames. This AM cycle was repeated 30 times (corresponding to a total trial duration of 11 ​s), each cycle being repeated at 2.6 ​Hz (Fig. 1A).

An additional 10 conditions (five at short-range and five at long-range condition) were presented to the participants in which no motion was perceived (Fig. 1B). These conditions were used to create predictions and to determine the contribution of spatial and temporal factors in the short- and long-range AM processing (see data analysis section 2.5). In the two *single conditions*, only one stimulus either the one on the left or the one on the right side of the fixation was presented. Each left or right stimulus was presented at the same frequency as in the AM condition (2.6 ​Hz). In the *simultaneous condition*, the left and right stimuli were flashed simultaneously at 2.6 ​Hz. In the *double conditions*, the stimulus was flashed at only one location but at double the frequency (5.2 ​Hz) either on the left or on the right side of the fixation. The experiment consisted in 18 blocks of 12 trials. In each block each condition was presented once in a random order.

In Experiment 2, we increased the strength of the motion percept by changing a few stimulus parameters. First, to avoid participants to fixate on the AM path, which weakens the AM percept (Kolers and von Grünau, 1977), the stimulus was smaller (1° ​× ​8° visual angle) and presented in the upper visual field, centred half-way between the top and the centre of the screen at around 7.5° visual angle. Second, we increased the duty-cycle (the ratio between the stimulus ON-time and the stimulus OFF-time) of the stimulus since the strength of AM increases with smaller SOA (Finlay and von Grünau, 1987; Grossberg and Rudd, 1992; Kolers and Pomerantz, 1971). This time, the stimulus was presented for 13 frames (153 ​ms) and disappeared for 4 frames (47 ​ms) (Fig. 1A). Thus, the AM cycle frequency, 2.5 ​Hz (5 ​Hz ​at each stimulus location), was almost the same as in Experiment 1 but the duty-cycle ratio was reversed. Finally, we also increased the eccentricity at which the stimulus was presented to 0.6° visual angle at short-range and 6° visual angle at long-range (the edge to edge spatial separation between the left and right stimulus was 0.2° and 11° for the short and long-range condition respectively). Each AM cycle was repeated 35 times in one trial for a total trial duration of 14 ​s. Experiment 2 consisted in 17 blocks of 12 trials, each trial corresponding to a different condition. The order of the trials was randomly chosen for each block.

### 2.3 Procedure

Participants were seated in a dimly lit room approximately 60 ​cm from the CRT screen. At the beginning of a trial, a fixation cross appeared at the centre of the screen. Participants were asked to keep fixating the cross as long as it was on the screen. They were also told to stay relaxed and try not to blink as much as possible to avoid noise in the EEG data. While the fixation was present, the stimulus was flashed periodically on the screen for 11 ​s in Experiment 1 and 14 s in Experiment 2. Because AM might rely on attentional mechanisms (e.g. Kohler et al., 2008; Lu and Sperling, 1995) we ensure that participants attended to the stimulus by asking them to report how many white dots (diameter of 0.2° and 0.5° visual angle in Experiment 1 and 2 respectively) appeared on the stimulus during the trial. They reported their response at the end of the trial by pressing the corresponding number on the computer numpad. The next trial started after participant’s response. There was no time limit to respond and participants were told that they could take a break before responding if they wished. They were also encouraged to take breaks between blocks.

The number of targets was 0, 1, 2 or 3, setting the level of chance at 25%. Participants’ performance was well above this chance level in all conditions with an average of 67.81% ​± ​3.99% (mean ​± ​SEM) detection rate in Experiment 1 and 88.78% ​± ​2.59% in Experiment 2. Participants were better in Experiment 2 most likely because the target dots were presented for the same duration as the stimulus, which was longer in Experiment 2 (153 ​ms) than in Experiment 1 (47 ​ms).

### 2.4 EEG pre-processing

EEG was recorded using a 128-channels BioSemi Active-Two system at 2048 ​Hz sampling rate. Two external electrodes were placed on each side of the eyes and another one below the right eye. Signal processing was conducted using FieldTrip (Oostenveld et al., 2011). Each trial was re-referenced to Cz, detrended and low-pass filtered at 85 ​Hz using a two-pass Butterworth (IIR) filter of order 6.

The data was then re-sampled at 510 ​Hz and for each trial, epochs of 2 ​s were created (discarding the first and last 1 ​s of a trial). This resulted in 72 epochs per condition in Experiment 1 (4 epochs per trial) and 102 epochs per condition in Experiment 2 (6 epochs per trial). Noisy channels (on average 6 per participant in both experiments) were replaced by the average signal of neighbouring channels. The data was then visually inspected to remove any epoch containing eye-blinks, saccades or excessive muscle activity. Following this procedure, on average 16% of epochs in Experiment 1 and 19% in Experiment 2 were rejected per participant. The number of rejected epochs was evenly distributed across conditions except in Experiment 2 at the long-range distance. After equating the number of rejected epochs, we found similar results as reported here.

In addition to the 2 ​s epochs, five sub-epochs of one AM cycle duration were created (corresponding to 376 ​ms in Experiment 1 and 400 ​ms in Experiment 2). These sub-epochs were noise filtered by only including the EEG response at the stimulus presentation frequency harmonics up to 50 ​Hz (2.66 ​Hz and its harmonics up to 47.81 ​Hz in Experiment 1 and 2.5 ​Hz and its harmonics up to 47.5 ​Hz in Experiment 2). These sub-epochs were averaged for each participant, electrode, and condition separately. These visual evoked potential waveforms corresponding to one AM cycle were used to create the EEG signal predictions (see below). This analysis is standard for periodic visual stimulation paradigms (Norcia et al., 2015).

### 2.5 EEG analysis

#### 2.5.1 Predictions

To determine the specific spatio-temporal EEG response to motion, we created ten different predictions (five for short-range and five for long-range). These can be summarised as follows:

1. a linear prediction was created from the linear summation of the EEG response to a right and a left signal
2. a spatial prediction was created in which spatial interactions were added to the linear prediction
3. a temporal prediction was created in which temporal interactions were added to the linear prediction
4. a spatial and temporal prediction was created including both types of interactions to the linear prediction
5. a regressed spatial and temporal prediction was created for which only a proportion of the spatial and temporal interactions was used in the prediction

By comparing these predictions to the observed AM response, we should be able to determine the contribution of the spatial and temporal interactions unrelated to motion. The signal not explained by these interactions should further indicate the specific motion component in AM. This component can then be compared for AM at short and long-range.

**Fig. 2:**
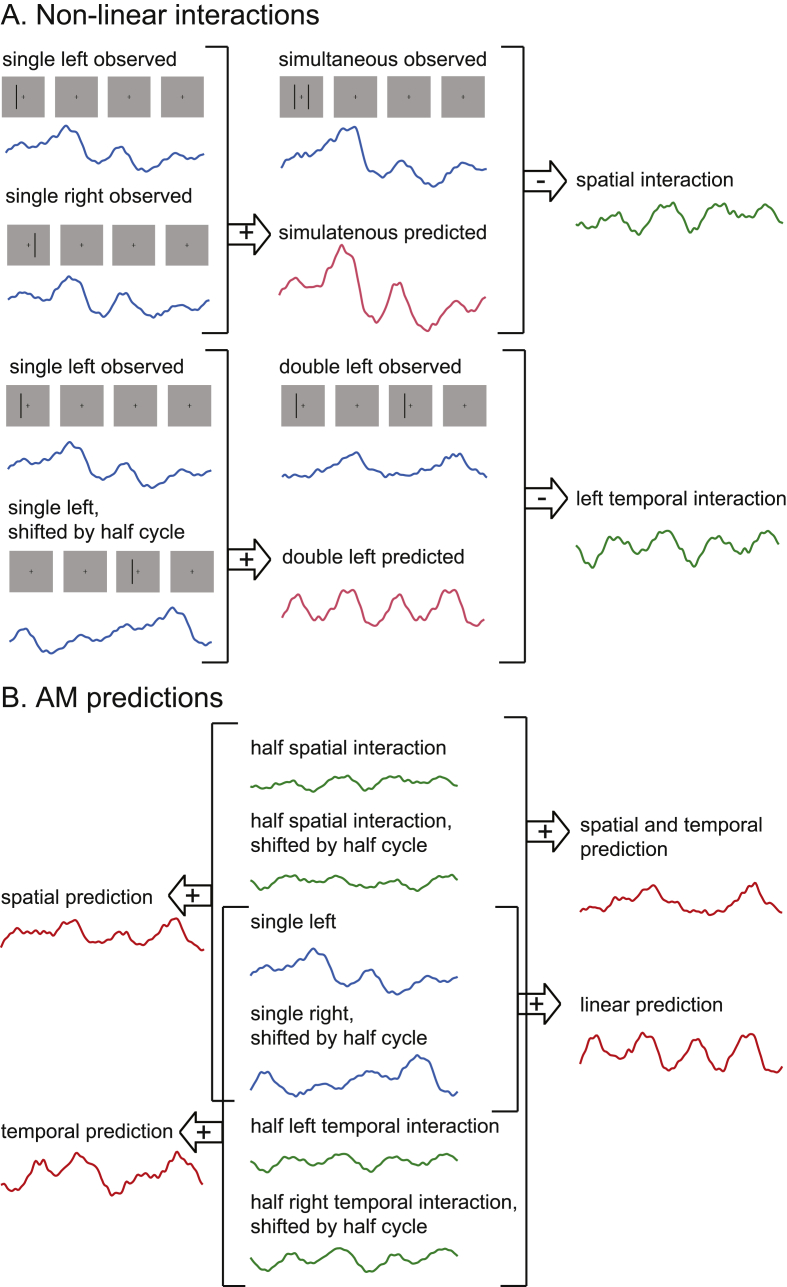
Illustration of the analysis steps implemented to predict AM. A. Analysis used to determine the spatial and temporal non-linear interactions (green EEG trace) unrelated to motion perception. The spatial interaction was determined from the difference between the observed (blue EEG trace) and the predicted (red EEG trace) simultaneous condition. The predicted simultaneous response was constructed from adding a single left and single right response. The left temporal interaction was determined from the difference between the observed and the predicted double left condition. The double left prediction was predicted from the addition of a single left and another single left response shifted by half an AM cycle. Only the left interaction is illustrated but the same steps were used for the right interaction using a single right response. B. The spatial, temporal, spatial and temporal, and the linear AM predictions (in red) were computed from the summation of responses observed in the single left and single right condition (in blue, central column) and the non-linear interactions (in green, central column) as shown.

We describe below how we determined the spatial and temporal non-linear interactions and how we constructed the ten different predictions. These steps are illustrated in Fig. 2 and the formal mathematical equations can be found in the Appendix. The same procedure was applied for short and long-range conditions. All the predictions were done using the pre-processed EEG sub-epochs of one AM cycle recorded in the different experimental conditions for each participant and electrode separately. In the results section the predictions are averaged across participants.

##### 2.5.1.1 Non-linear interactions

Non-linear *spatial* interactions were computed from the difference between the EEG signal recorded in the simultaneous condition and a predicted simultaneous response created from the linear summation of the left and right single stimulus condition (eq. (A.2), Fig. 2A).

The non-linear left and right *temporal* interactions were created by computing the difference between the EEG response to the double left (or right) condition and a predicted double left (or right) condition (eq. (A.4), Fig. 2A). The predicted double left (or right) response was generated from the summation of the observed single left (or right) response and the observed single left (or right) response shifted by half an AM cycle in time.

##### 2.5.1.2 Linear prediction

For the linear prediction, we summed the signal recorded in the left and right single stimulus conditions (eq. (A.1), Fig. 2B). However, because the stimulus was presented at the beginning of the epoch in both single conditions, one response had to be shifted half an AM cycle in time to match the AM sequence (presentation of a left stimulus followed by a right stimulus).

##### 2.5.1.3 Spatial prediction

The spatial prediction of AM was created from the summation of a left and right response which included a spatial interaction (eq. (A.3), Fig. 2B). The response to the left stimulus was predicted by summing the left single response with half the spatial interaction. The response to the right stimulus was predicted by summing the right single response with half the spatial interaction, both signals being shifted half an AM cycle in time to match the AM sequence.

##### 2.5.1.4 Temporal prediction

For the temporal prediction we summed the observed left single response, the computed left interaction, the observed right single response shifted half cycle in time and the computed right interaction shifted half cycle in time (eq. (A.6), Fig. 2B). This temporal prediction represents therefore the EEG response for an AM sequence with temporal interactions but no motion percept.

##### 2.5.1.5 Spatial and temporal prediction

The spatial and temporal prediction was created by adding the left and right single response which included both interactions. The left response was created by adding a single left response with half a spatial interaction and half a left interaction. The right response was created similarly by adding a single right response with half a spatial interaction and half a right interaction. The signals used to create the right response were all shifted half an AM cycle in time so that it would mimic the AM sequence. These left and right responses were then summed to create the spatial and temporal prediction (eq. (A.7), Fig. 2B).

##### 2.5.1.6 Regressed prediction

We assumed that AM was the summation of the EEG response to a left and a right stimulus with spatial, temporal and spatio-temporal interactions. However, it is possible that only a proportion of the spatial and temporal interactions unrelated to motion are involved in the AM response. To test this possibility, we applied a linear regression and determined the spatial and temporal regression coefficients for which we could find the best fit to the observed AM. The regression was set considering both non-linear interactions but only one spatial and one temporal coefficient was determined for all electrodes and all time-points for each participant independently (i.e. two regression coefficients were determined per participant). These coefficients were then used to create a regressed prediction. This prediction was computed the same way as the spatial and temporal prediction but by applying the spatial coefficient to the spatial interaction and the temporal coefficient to the temporal interaction (eq. (A.8)).

#### 2.5.2 Prediction error

To assess how close each prediction was to the AM signal, we calculated the difference between each prediction and the AM signal, and computed the root-mean-square (RMS) of this difference across time for each electrode separately (eq. (A.9)). This root-mean-square error (RMSE) can be thought of as the excess standard deviation of the amplitude difference between the observed and predicted EEG response. In other words, the RMSE represents the amount of signal that is still present in the difference between the predicted and the observed response such that the higher the RMSE is, the more signal not accounted by the prediction there is. By visualising the topography of the RMSE we hoped to determine if there was a specific brain area for which we could not predict the AM response. This could therefore be the one from where motion signal originates.

However, the RMSE includes both the signal present in the prediction error and the signal from the background noise EEG activity. Importantly, the background EEG activity was not the same in each condition and was higher for some predictions compared to others due to the propagation of error. For example, the noise level would be higher in the spatial than the linear prediction since for the spatial prediction, the noise from the simultaneous condition would also be present (in addition to the noise from the single left and single right conditions). Therefore, in order to more easily compare across these conditions, we calculated a normalised RMSE (NRMSE) for each of the prediction by taking the ratio between the RMSE and the RMS of the estimated noise variance in the signal (eq. (A.10)). Since we used a periodic visual stimulation, we could estimate the background EEG activity (or noise activity). Here, we averaged the amplitude of the two noise frequencies adjacent to the frequency at which the AM stimulus was presented (2.2 ​Hz and 3.1 ​Hz in Experiment 1, 2 ​Hz and 3 ​Hz in Experiment 2) to estimate the amplitude of the noise activity at the AM frequency (2.6 ​Hz and 2 ​Hz in Experiment 1 and 2 respectively). The same procedure was applied for the other frequencies present in the stimulus presentation (all the harmonics) to estimate a noise response for the observed AM and in each of the prediction (eq. (A.11)). This estimated noise amplitude was then used for determining the NRMSE for each prediction (see also eq. (A.12) and eq. (A.13)).

To quantify the prediction error compared to the observed AM response, we calculated the inverse of the NRMSE (1/NRMSE) which corresponds to the proportion of the prediction error that is equal to the noise level. A value of 1 represents the noise ceiling, that is, the amplitude difference between the observed and predicted responses is the same as the noise level when no evoked signal is present.

Thanks to normalising the RMSE, we could directly compare the predictions with each other. We compared all the short-range predictions with each other and all the long-range predictions with each other using Wilcoxon sign-rank tests. All p-values are reported after Bonferroni correction (for 10 comparisons).

Another way to test the goodness of fits would be to determine the amount of signal that is explained by the different predictions. However, this measure does not take into account the strength of the observed signal that needs to be explained. For example, in our study, since the observed response was larger in Experiment 1 than in Experiment 2, the explained signal variance could be larger in Experiment 2 just because there was less signal to predict. Therefore, determining the nature of the residuals as we did is a better measure to assess the fit of our predictions.

## 3 Results

### 3.1 Observed AM response

**Fig. 3:**
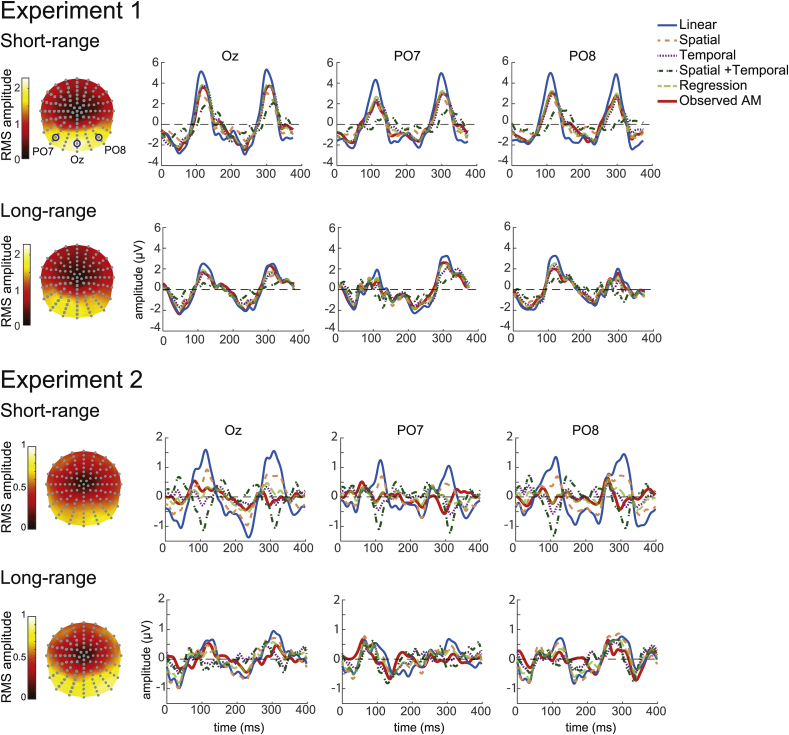
Observed and predicted AM response in Experiment 1 and 2 ​at short and long-range distances. The topographies (left column) represent the RMS amplitudes calculated for each electrode in the observed AM. The EEG waveforms for the observed and predicted responses during one AM cycle are represented for the three posterior electrodes indicated in the top left topography. Note that the scale is different between the two experiments.

To get a general idea of the topography of the brain response during AM at short and long range, we computed the total signal strength (RMS) over time for each electrode. This value represents the amount of the EEG signal deviation from 0. As expected, most of the response to the stimulus was located at the posterior electrodes (Fig. 3, left column). The topographies were in general very similar for short and long-range AM. However, the RMS were much lower, by a factor of 2, in the second experiment. This difference was also visible in the visual evoked potential (VEP) waveforms illustrated for three electrodes (a central, Oz, and two lateral electrodes, PO7 and PO8) in Fig. 3. We discuss possible explanation for this observation in the discussion (section 4.3).

The VEP waveforms represent the brain response over time for the presentation of a left and a right stimulus (one AM cycle). The presentation of these successive stimuli generates two peaks at around 120 and 300 ​ms. One can note that the amplitude of these two peaks was more asymmetrical at long than at short-range for PO7 and PO8 (Fig. 3). That is, the brain response was more lateralised in the long-range condition. This is not surprising given that the stimuli were presented further away from the central fixation at long-range.

### 3.2 Predicted AM response

The aim of this study was to determine a response to AM stimuli which controls for both spatial and temporal non-motion specific interactions. In order to establish the specific spatio-temporal response underlying the perception of motion, we estimated the linear component of the response to AM from a linear summation of a left and right response. We also estimated the non-linear interactions created from purely spatial and purely temporal non-linearities, and the combination of these interactions. It is important to point out that these interactions are generated by mechanisms that are not tuned to motion.

In the short-range condition of Experiment 1, in all three reported electrodes (results are similar at other electrode locations), visual inspection indicates that the linear prediction has a higher amplitude than the response observed for AM (Fig. 3, top row). This would suggest that in the AM condition, the brain response is inhibited. Interestingly, the spatial and particularly the temporal predictions look very close to the observed AM. Thus, the spatial and temporal interactions taken separately might have an inhibitory effect important for predicting the AM response. However, even though AM is defined by its spatio-temporal pattern, combining spatial and temporal interactions did not seem to improve the predicted response. This suggests that the inhibitory spatial and temporal interactions might be so similar with one another that their sum would generate too much inhibition. Indeed, if regression coefficients were applied to the spatial and temporal interactions, the prediction seems to fit the observed AM better.

At long-range, apart from the combination of spatial and temporal interactions, all the other predictions visually matched the observed AM well (Fig. 3, second row). Spatial, temporal and the regressed predictions were very similar to each other and appeared to diverge only slightly from the observed AM at around 50, 250, and 300 ​ms when considering the electrode Oz. The linear prediction also looked very similar to the observed AM but deviated from it at a different time, around 110 ​ms. Therefore, at long-range distances, spatial and temporal interactions did not appear to be important to predict the AM response. However, just as for short-range, the combination of spatial and temporal interactions did not seem to improve the AM prediction.

Concerning Experiment 2, as previously reported, the VEP amplitudes were at least two times lower than in Experiment 1 (Fig. 3, note the amplitude scale difference between Experiment 1 and 2). However, the pattern or results was relatively similar to Experiment 1. After visual inspection, we observe that at short-range distance, adding spatial or temporal interactions to the linear prediction appeared to inhibit the signal such that the predictions looked closer to the observed AM. This was the case especially for the temporal prediction. However, the combination of both interactions did not seem to improve the prediction unless regression coefficients were applied to those interactions. At long-range distances, the observed and predicted responses visually appeared very similar to each other, but they were also very small in amplitude. Thus, controlling for spatial or temporal interactions did not seem to improve the AM predictions.

In summary, to our surprise, AM predictions appeared very close to the observed AM. That is, most of the AM response seemed to be explained by either a linear summation at long-range or by the addition of temporal interactions not specific to motion at short-range. This also means that the spatio-temporal interaction specific to motion that we aimed to find might be very small (if not inexistent) in our study. In the next section we determined more formally the amount and the nature of the brain response not accounted by our AM predictions.

### 3.3 Prediction error

**Fig. 4:**
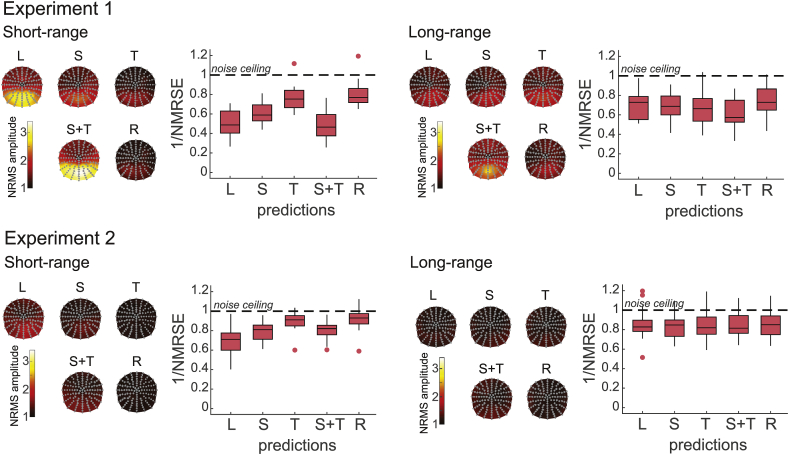
Prediction error in Experiment 1 and 2 ​at short (left) and long-range (right) distance for the linear (L), spatial (S), temporal (T), spatial and temporal (S ​+ ​T) and regressed spatial and temporal (R) predictions. The topographies represent the amplitude of the normalised residuals (NRMSE) averaged across participants. The proportion of the prediction error (1/NRMSE) to the residual EEG activity (noise ceiling) averaged across electrodes is illustrated in the boxplots. The closer this value is to the noise ceiling, the closer the prediction is to the observed AM signal. The bottom and top edges of the boxes indicate the 25th and 75th percentiles while the central mark indicates the median. Datapoints outside 1.5 times the interquartile range are symbolized by individual dot and are treated identically to other datapoints.

To formally compare how well the predictions fit the observed AM response, we computed the root-mean-square (RMS) of the amplitude difference (residuals or prediction error) between the observed and predicted response normalised by the estimation of the noise variance (normalised root-mean-square error, NRMSE; see Methods section 2.5.2 for more details). We first computed the NRMSE over all time points for each electrode separately to look for any specific location with a high NRMSE, that is, with the largest unexplained stimulus response. Such location would reflect a brain region responding specifically to a spatio-temporal interaction underlying motion perception. However, most of the difference between the observed and predicted AM was not restricted to one area but distributed over a large set of posterior electrodes in both experiments and for all types of predictions (Fig. 4, topographies). Thus, given that the NRMSE topographies were similar across predictions, we computed the NRMSE over all electrodes and obtained a single value per prediction and participant.

A prediction never matches the observed data perfectly because there is always residual noise in the data. In fact, a perfect match might give a hint that the data has been overfitted. By using the NRMSE, we can obtain a measure of fit quantified in terms of residual noise (proportion of noise contained in the residuals). We used here the inverse NMRSE which reflects the goodness of fit between the predictions and the observed AM. A high value of NRMSE (close to 1, 100%) means that the prediction is very close to the observed AM response (the observed amplitude difference is the same as the noise level) while a low value means that the residuals still contain a strong signal. The inverse NRMSE is therefore a quantification of the EEG results described in the previous section.

In Experiment 1, at short-range, the linear, spatial and the combination of spatial and temporal predictions were the ones furthest from the noise ceiling that is, with the most residual signal (Fig. 4). The prediction error was similar (on average 54% of the residual error can be attributed to noise) across these three predictions (all Z<2.27, p>0.23). On the other hand, the inverse NRMSE of the temporal and the regressed predictions were around 79% that is, the predictions were closer to the noise ceiling compared to the other linear, spatial and the combination of spatial and temporal predictions (all Z ​> ​2.76, p ​< ​0.06). There was no difference between the amount of residual signal for the temporal and the regressed prediction (Z ​= ​2.48, p ​= ​0.13). Thus, temporal interactions were important in predicting short-range AM and the residual signal was negligible when these interactions were accounted for. At long-range in Experiment 1, controlling for spatial and temporal interactions did not improve the prediction fit to the observed AM compared to a linear summation (all Z<2.13, p>0.33). The prediction error was relatively low in all conditions (on average the majority of the residual, 68%, was at the noise level) and there was no difference across predictions (all Z<2.27, p>0.23) apart from a slight improvement for the regressed prediction compared to the temporal prediction (Z ​= ​2.69, p ​= ​0.07) and to the combination of spatial and temporal predictions (Z ​= ​2.97, p ​= ​0.03). Thus, the role of spatial and temporal interactions was minimal in predicting long-range AM and a simple linear summation already gave a good estimate of the AM response.

In Experiment 2, we observed the same pattern of results (Fig. 4). At short-range, the residual signal was closer to the noise ceiling in the temporal and the regressed predictions compared to the other predictions (all Z ​> ​3.01, p ​< ​0.03). A similar amount, around 91%, of the residuals was at the noise level in the temporal and regressed predictions (Z ​= ​1.30, p ​> ​0.99). One difference with Experiment 1 is that although the inverse NRMS was lower for the linear compared with the spatial prediction in both experiments, this effect was significant only in Experiment 2 (Z ​= ​3.01, p ​= ​0.03). In sum, as in Experiment 1, temporal interactions were important in predicting short-range AM. On the other hand, at long-range, on average 84% of the residual signal was at the noise level and there was no difference across all predictions (all Z<1.35, p>0.99). Thus, long-range AM can be well predicted by a linear summation of independent responses to a left and a right stimulus.

Consistent with the observations made in the previous section on the EEG waveforms, AM can be well predicted from brain responses to non-motion signals. Between 68% and 91% of the difference between the prediction and the observed AM was at the level of residual noise. Thus, in our study, a specific spatio-temporal response to motion was almost inexistent. However, it is interesting that although the role of non-linear interactions was minimal in long-range, temporal interactions were essential in predicting short-range motion in both experiments.

### 3.4 Non-linear interactions

**Fig. 5:**
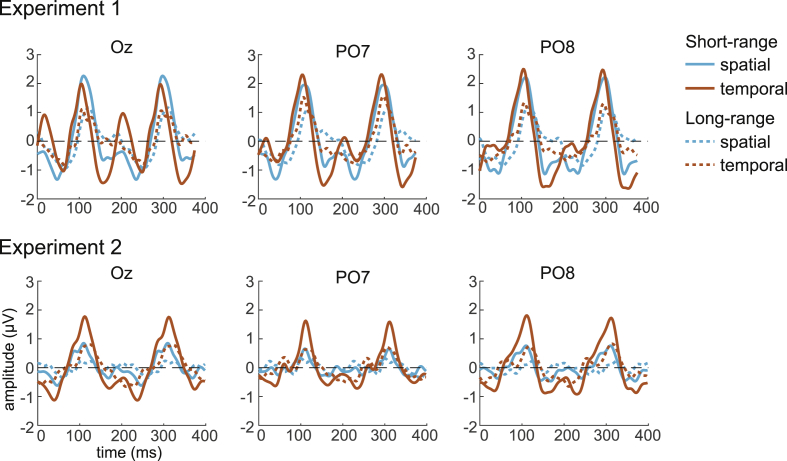
Non-linear spatial (blue) and temporal (red) interactions at three posterior electrodes in Experiment 1 (top row) and 2 (bottom row) for short-range (plain lines) and long-range (dashed lines).

Our results show that adding both spatial and temporal interactions did not improve the fitting with the AM response but instead was detrimental. It seems therefore plausible that these two interactions were very similar. The non-linearity determined for spatial and temporal interactions are represented in Fig. 5. In Experiment 1, these non-linearities were larger at short than long-range but relatively similar between spatial and temporal interactions with two peaks at around 100 and 300 ​ms. In Experiment 2, the temporal interaction at short-range distance has the strongest amplitude with peaks at around the same time (100 and 300 ​ms) as in Experiment 1, while other interactions are two times smaller. Thus, although the amplitude of non-linearities contained in the signal is different across conditions, their waveforms are very similar. Surprisingly, this similarity applies between spatial and temporal interactions, between short and long-range and also between Experiment 1 and 2. This might be the consequence of our paradigm in which the stimuli are presented at a particular frequency. This periodic stimulation might emphasise the brain response to the stimulus presentation frequency (and frequency harmonics) which would then generate non-linear interactions at similar frequencies. In any case, the similarity between the non-linear interactions can certainly explain why the contribution of temporal and spatial interactions was detrimental in predicting AM: their combination would yield to multiplying one interaction by a factor of two.

Another point of importance is that non-linear interactions were around half the amplitude of the observed AM signal at both short and long-range, yet non-linear interactions were essential in predicting AM only at short-range. In other words, spatial and temporal interactions at long-range were present but did not improve the AM prediction. Thus, the minimal role of non-linear interactions at long-range is not the result of an absence of interactions. Instead, our results suggest that short and long-range AM rely on different neural mechanisms.

## 4 Discussion

In this study, we aimed to determine and compare the specific motion brain response generated during short and long-range AM by carefully controlling for spatial and temporal interactions that were not related to a moving stimulus. We conducted two experiments with either a weaker (Experiment 1) or stronger (Experiment 2) motion percept during AM and found a similar pattern of results in both cases, although the EEG signal was much lower in Experiment 2. At short-range, inhibitory non-linear interactions and particularly temporal interactions, were essential in predicting the EEG responses to AM. After controlling for these temporal interactions, 79% and 91% of the residual error was attributable to the noise level in the EEG recording in Experiment 1 and 2 respectively. At long-range, the linear prediction was as good in predicting AM as the predictions controlling for spatial and temporal interactions, with 68 and 84% of the residual signal at the level of noise. Thus, we could predict AM from non-motion related EEG response very well, implying that a specific motion related response was absent or very weak in the neural responses that we recorded.

In our approach, we compare an observed response to a predicted one. This allows us to determine that at short-range, temporal interactions are very important in predicting AM responses. Importantly, this approach demonstrates that the difference between the brain response to AM and the linear summation of a single left and a single right response cannot be considered as the substrate of “motion processing”. Previous experiments only controlled for spatial interactions (Norcia et al., 2017; Wibral et al., 2009) and might have therefore reported a motion response that would have included a temporal non-linear interaction. Here we show that after controlling for both spatial and temporal interactions, AM can be fully accounted for by responses to non-moving stimuli. There is no specific neural response to motion, at least in set-ups similar to ours. Another benefit of our method is that it allows comparisons across experiments. Because it is based on the goodness-of-fit between a prediction and an observed response, the initial strength of the response does not influence the results. It is independent of the specific experimental parameters used in a study.

### 4.1 Absence of a spatio-temporal EEG signature of motion

By controlling for non-motion related spatial and temporal factors, we expected to find a spatio-temporal signature of motion perception in AM. However, we did not find this signature in either short- or long-range motion. Instead, the AM response was dominated by non-linearities not specific to motion processing. One possibility is that the motion strength was too weak to evoke an observable difference in the EEG response. However, we believe that it is not the case for several reasons. First, although AM might be weaker in the long-range AM conditions, it should be evident at short-range. That is, we might not find a spatio-temporal signature of motion at long-range, but we should be able to find one at short-range. Instead, there was no sign of a motion specific EEG response at either short or at long range. Further, despite our efforts to increase the motion strength in Experiment 2, the pattern of results was remarkably similar between the two experiments. Stimulus parameters that are known to induce a stronger AM percept (Finlay and von Grünau, 1987; Grossberg and Rudd, 1992; Kolers and Pomerantz, 1971; Kolers and von Grünau, 1977) did not influence the neuronal responses and no purely motion-related EEG response was found in any condition. Finally, the perception of AM over other percepts such as flickering stimuli mainly depends on temporal parameters and we chose temporal parameters for which AM is usually reported (Anstis et al., 1985; Finlay and von Grünau, 1987; Kolers, 1972; Kolers and Pomerantz, 1971; Kolers and von Grünau, 1977; Miller and Shepard, 1993). Specifically, in a thorough study, Ekroll and colleagues (Ekroll et al., 2008) show that for the temporal parameters that we used (200 ​ms SOA and positive duty-cycle), participants report seeing AM; they never report a flicker or an appearance-disappearance percept. Furthermore, the authors presented the two stimuli around 2.5° of visual angle apart from each other, which is close to the 3° separation we used in Experiment 1 at long-range. Thus, we believe that participants perceived motion in the AM conditions of our study. The absence of an EEG signature of motion in all AM conditions cannot be explained by an absence of a motion percept.

In our study, AM was presented across hemifields which might affect neural responses compared to an AM stimulus presented within a single hemifield (as in Wibral et al., 2009). Indeed, previous studies have shown the existence of a vertical motion bias when participants had to fixate at the centre of a motion quartet (Chaudhuri and Glaser, 1991; Kohler et al., 2008). That is, participants report seeing vertical motion more often than horizontal motion. Note however that this bias does not influence the perception of AM when only one stimulus is presented alternatively on the left and right side of the fixation (e.g. Ekroll et al., 2008; Kolers and von Grünau, 1977). The vertical motion bias seems to be the result of a delay in the transmission through the corpus callosum (Genç et al., 2011) which might indicate differences in intra or inter-hemispheric motion processes. However, apart from a delay in the integration process, it would be surprising if the brain processed motion differently within and across hemifields. We cannot rule out this possibility but previous studies have reported the involvement of similar brain regions (such as MT) during AM compared to a flickering percept regardless of whether the motion was intrahemispheric (Akselrod et al., 2015; Muckli et al., 2005; Wibral et al., 2009), interhemispheric (Muckli et al., 2002; Sterzer et al., 2006; Zhuo et al., 2003), concentric (Liu et al., 2004) or originating from illusory contours or mental imagery (Goebel et al., 1998). Hence it is unlikely that presenting the stimuli across hemifields can account for the lack of a motion specific signal. Importantly, whether inter and intra-hemispheric motion rely on the same or different processes does not affect our conclusions.

Another possibility is that the motion specific response that we are looking for is not located in the occipital cortex but in other processing areas that might be difficult to detect using our set-up. Many cortical areas other than visual areas respond to motion (Sunaert et al., 1999) and whereas information about the physical position of an object is represented in early visual areas, its conscious or perceived position seem to be represented in higher brain regions (Fischer et al., 2011; Liu et al., 2019). That is, there seems to be a dissociation between the perceived position of an object and the neural activity it generates in visual areas. In light of these findings, it might not be too surprising that the neural responses we recorded, mostly visual, do not contain information about motion perception per se. Nevertheless, it is impressive that we could predict AM response so well from non-motion related responses. In fact, such results highlight the relevance of our methodology.

### 4.2 EEG responses at short and long range

One goal of our study was to compare the neural response to AM at short and long-range distances. The difference between short and long-range AM has been debated previously (Burr and Thompson, 2011; Cavanagh and Mather, 1989; Lu and Sperling, 2001; Von Grünau, 1986) but the distinction we make here is about the spatial separation between the two objects. Specifically, whereas models of visual motion and electrophysiological results support the existence of neurons responding to short-range motion, there is no such support for long-range motion (Adelson and Bergen, 1985; Churchland et al., 2005; Livingstone et al., 2001; Rust et al., 2006; Simoncelli and Heeger, 1998). Therefore, we tested whether EEG responses would be similar or different for short and long-range AM. Thanks to our methodology based on predicting the AM response, we could compare the role of spatial and temporal interactions at short and long range. We did not find a specific spatio-temporal response related to motion perception in our experiments. However, our results show that spatial and temporal non-linearities unrelated to motion in the EEG signal are more important at short than at long range. This might be because the neuronal population responding to the left and right stimuli are closer to each other at short than at long-range, and would thus interact more. In any case, the spatial separation between the two stimuli influences the brain response, suggesting that the short and long-range processes are based on different neural mechanisms.

Contrary to our findings, Norcia et al. (2017) did not report a difference in the role of non-linear interactions at short and long-range. In both distance conditions, they found that EEG responses for AM was smaller than a linear prediction. After adding non-linear spatial interactions to the linear prediction (they did not correct for temporal interactions), the AM prediction improved by a factor of 1.5. These results are similar to what we observed in our study for short-range but not for long-range AM. Although the two studies use different stimuli (vertical bars vs. gratings), eccentricity and stimulus-onset-asynchrony and are thus not directly comparable, the spatial separation between the two stimuli were quite different. Norcia et al. used stimuli that were vastly overlapping, with a spatial offset of only 0.125° in the short-range condition and 1.5° in the long-range condition (0.5° edge-to-edge) while our stimuli were separated by a larger distance, 0.6° at short-range and 6° at long-range (1.2° and 12° in Experiment 2). The exact spatial distance at which a stimulus is considered short-range vary between studies and also depends on other parameters such as the eccentricity of the stimulus or the stimulus-onset-asynchrony (Baker and Braddick, 1985; Churchland et al., 2005; Larsen et al., 1983; Pack et al., 2006). In general, the short-range process has been defined for successive dots separated by less than 1° in space and less than 100 ​ms in time. In addition to differences in spatial distance, in Norcia et al.’s study 13 stimuli were presented simultaneously with some that were up to 16° away from the fixation. Thus, it is possible that the long-range condition of their experiment might have included some short-range neural responses, which would explain why their results are similar at short and long-range.

### 4.3 Differences in EEG response between the two experiments

Another important finding of our study is that although the results are similar between the two experiments, the brain response to AM was smaller in Experiment 2 than in Experiment 1. The group of participants was different between the two experiments, but we think that it is unlikely that such a strong effect could be explained by a difference in the individuals that we tested. Many stimulus parameters were modified in Experiment 2 to induce a stronger AM percept. Therefore, the inhibition of the neural responses that we observe in Experiment 2 could be due to the higher strength of the motion percept. Indeed, the brain response to moving stimuli could be inhibited because of their predictability (Rao and Ballard, 1999). It has also been suggested that motion could inhibit neural responses on the AM path (Alink et al., 2010; Van Humbeeck et al., 2016). However, the AM predictions were correspondingly smaller in the second experiment. That is, the EEG response was inhibited even for non-moving stimuli (e.g. the linear summation of a left and right single stimulus response was smaller in Experiment 2 although no motion was perceived). Consequently, it is not the motion percept that inhibited the neural response but the stimulus parameters that we changed between the two experiments.

Four parameters were modified in Experiment 2: the eccentricity, the location, the size, and the duty-cycle of the stimulus. The role of eccentricity on EEG responses can be assessed by comparing the short and long-range conditions. If we consider the electrode Oz in Experiment 1, its maximum amplitude at short-range (0.3° eccentricity) reduced by half at long-range (3° eccentricity). In Experiment 2, the maximum Oz response at short-range (0.6° eccentricity) reduced even more than what we observe at long-range in Experiment 1. This suggests that eccentricity cannot be the sole or primary factor contributing to the dramatic decrease in the EEG responses in Experiment 2. In addition, in Experiment 2 we presented the stimulus above the fixation (instead of across the fixation) and the total area of the stimulus was smaller (1° ​× ​8° in Experiment 2 vs. 0.5° ​× ​20° in Experiment 1). Together these modifications might have reduced the activation of the visual cortex during the presentation of the stimulus in Experiment 2 (Busch et al., 2004; Jeffreys et al., 1992; Luck and Kappenman, 2012; Rousselet et al., 2005). A final parameter that we have modified is the duty-cycle which increased from 25% in Experiment 1–75% in Experiment 2. To our knowledge the effect of duty-cycle on EEG response has not been tested systematically. Most of neuroimaging studies on AM have used a 50% duty-cycle (e.g. Norcia et al., 2017) and it might be possible that the increase in duty-cycle decreases brain responses. Indeed, VEP amplitudes are sensitive to the specific time at which a following stimulus is presented. This has been shown in masking paradigms (Polat et al., 2007) but also when the target visibility is not affected (van Aalderen-Smeets et al., 2006). In these studies, the VEP amplitudes of two successive stimuli are smaller when the inter-stimulus-interval is smaller since the VEP of the two stimuli overlap more. Thus, in our study, we would expect smaller amplitude response with longer duty-cycle (corresponding to a smaller inter-stimulus-interval), which is what we found. In summary, any of the modification done to the stimulus can explain the decrease in brain response between the two experiments. However, the inhibition related to these modifications cannot be explained by the perception of motion as the inhibition was present in the AM condition but also in non-motion conditions.

## 5 Conclusion

In two different experiments, we investigated the neural mechanisms underlying AM at short and long-range. We found that while inhibitory temporal interactions unrelated to motion are important in predicting short-range AM, non-linear interactions do not actually improve the predictions for long-range AM. This supports the idea that short- and long-range processes rely on different neural mechanisms. Importantly, for both short- and long-range, the AM predictions created from the EEG response to static stimuli were very close to the recorded EEG in response to moving stimuli. That is, there was no trace of a specific neural signature of motion perception. The brain response to AM is a simple combination of responses to independent non-moving stimuli.

## CRediT authorship contribution statement

**Marlene Poncet:** Conceptualization, Investigation, Methodology, Formal analysis, Writing - original draft, Writing - review & editing. **Justin M. Ales:** Conceptualization, Funding acquisition, Formal analysis, Methodology, Supervision, Writing - review & editing.

## Appendix

### AM predictions

In all equations, Y represents a 3-dimensional matrix containing the EEG amplitude at all timepoints for all electrodes and participants. Y is the observed EEG data while Ŷ is the predicted EEG response.

#### Linear prediction

The linear prediction was defined as:

(1) $$\hat{Y_{t}^{L}}=Y_{t}^{S1}+Y_{t+n}^{S2}$$

Where Y_t_^S1^ is the observed signal in the single left condition and Y_t+n_^S2^ is the observed signal in the single right condition shifted half cycle in time (*n*) to match the AM sequence.

#### Spatial prediction

The spatial prediction was constructed from the linear prediction with the addition of non-linear spatial interactions. Thus, we first had to determine the non-linear spatial interactions. We did so by comparing the EEG response observed in the simultaneous condition (Y_t_^SIM^) with a prediction of this simultaneous response using the single left and right condition:

(2) $$W_{t}^{S}=\left(Y_{t}^{S1}+Y_{t}^{S2}\right)-Y_{t}^{SIM}$$

Where $W_{t}^{S}$ represents the spatial non-linear interaction. The spatial prediction was then calculated by summing the observed response for a left and a right response including half a spatial interaction (note that the right response is shifted half AM cycle in time):

(3) $$\hat{Y_{t}^{S}}=\left(Y_{t}^{S1}+\frac{W_{t}^{S}}{2}\right)+\left(Y_{t+n}^{S2}+\frac{W_{t+n}^{S}}{2}\right)$$

#### Temporal prediction

The non-linear temporal interactions were calculated separately for the right and the left stimulus but using the same procedure in both cases. First, a predicted signal was created from the linear summation of a single stimulus response with the same single stimulus response shifted half a cycle in time. The difference between this predicted double condition and the recorded signal in the double condition was then calculated to obtain the non-linear temporal interactions for the left and the right stimulus:

(4) $$W_{t}^{T1}=\left(Y_{t}^{S1}+Y_{t+n}^{S1}\right)-Y_{t}^{D1}$$

(5) $$W_{t}^{T2}=\left(Y_{t}^{S2}+Y_{t+n}^{S2}\right)-Y_{t}^{D2}$$

Where Y_t_^D1^ is the observed signal in the double condition for a stimulus presented on the left, Y_t_^D2^ is the observed signal in the double condition for a stimulus presented on the right. The non-linear temporal interactions (W_t_^T1^ and W_t_^T2^) were then added to the observed signal in the single right and single left conditions to create a predicted AM signal that accounts for temporal interactions (note again that the response to the second stimulus is shifted half AM cycle in time):

(6) $$\hat{Y_{t}^{T}}=\left(Y_{t}^{S1}+\frac{W_{t}^{T1}}{2}\right)+\left(Y_{t+n}^{S2}+\frac{W_{t+n}^{T2}}{2}\right)$$

#### Spatial and temporal prediction

The prediction incorporating both spatial and temporal non-linear interactions was computed by including both spatial and temporal non-linear interactions to the observed responses to the single right and left conditions:

(7) $$\hat{Y_{t}^{S+T}}=\left(Y_{t}^{S1}+\frac{W_{t}^{S}}{2}+\frac{W_{t}^{T1}}{2}\right)+\left(Y_{t+n}^{S2}+\frac{W_{t+n}^{S}}{2}+\frac{W_{t+n}^{T2}}{2}\right)$$

#### Regressed prediction

In addition to the spatial and temporal prediction we also included the possibility that only a proportion of the spatial and temporal interactions were involved in predicting AM. Two regression coefficients, *a* for the spatial interaction and *b* for the temporal interaction, were added to the previous equation. Note that the same coefficients were used across timepoints and electrodes but were different for different participants.

(8) $$\hat{Y_{t}^{R}}=\left(Y_{t}^{S1}+a\times \frac{W_{t}^{S}}{2}+b\times \frac{W_{t}^{T1}}{2}\right)+\left(Y_{t+n}^{S2}+a\times \frac{W_{t+n}^{S}}{2}+b\times \frac{W_{t+n}^{T2}}{2}\right)$$

### Prediction error

To estimate the goodness-of-fit of the EEG predictions, we computed the root-mean-square-error (RMSE) between a given prediction condition (*C*^P^) and the observed AM EEG response (*C*^AM^):

(9) $$RMSE_{n}^{P}=\sqrt{\frac{\sum_{t=1,e=1}^{T,E}{\left({\hat{C}}_{e,t,n}^{P}-C_{e,t,n}^{AM}\right)}^{2}}{TE}}$$

Where *e* represents an electrode (E the total number of electrodes), *t* represents one timepoint (T the total number of timepoints) and *n* a participant number. The RMSE was calculated for each prediction (linear, spatial, temporal, spatial and temporal, regressed) and each participant separately.

The normalised-root-mean-square-error (NRMSE) was computed by dividing the RMSE of a given prediction with the baseline noise variance (RMS) contained in the observed AM (B^AM^) and in the prediction (B^P^) (see Method for how the baseline noise was determined):

(10) $$NRMSE_{n}^{P}=\frac{RMSE_{n}^{P}}{\sqrt{RMS{\left(B_{n}^{AM}\right)}^{2}+RMS{\left(B_{n}^{P}\right)}^{2}}}$$

Where the baseline RMS is calculated for each condition and each participant separately as follows:

(11) $$RMS\left(B_{n}^{C}\right)=\sqrt{\frac{\sum_{t=1,e=1}^{T,E}{\left(B_{e,t,n}^{C}\right)}^{2}}{TE}}$$

Importantly, the spatial and temporal prediction includes baseline noise from the spatial and the temporal interactions. The RMSE for the spatial and temporal prediction needs therefore to be divided by the baseline noise variance from the AM prediction (B^AM^), the spatial prediction (B^S^) and the temporal prediction (B^T^):

(12) $$NRMSE_{n}^{S+T}=\frac{RMSE_{n}^{S+T}}{\sqrt{RMS{\left(B_{n}^{AM}\right)}^{2}+RMS{\left(B_{n}^{S}\right)}^{2}+RMS{\left(B_{n}^{T}\right)}^{2}}}$$

Similarly, in the regressed prediction, the amount of baseline noise variance from the spatial and temporal predictions is dependent on the regression coefficient (*a* and *b*). Thus, the NRMSE for the regressed prediction is calculated as:

(13) $$NRMSE_{n}^{R}=\frac{RMSE_{n}^{R}}{\sqrt{RMS{\left(B_{n}^{AM}\right)}^{2}+a\times RMS{\left(B_{n}^{S}\right)}^{2}+b\times RMS{\left(B_{n}^{T}\right)}^{2}}}$$

## Data and code availability statements

Original behavioural data and processed EEG data are made publicly available on OSF as well as the Matlab programs used to run the experiments and to analyse the data (DOI:10.17605/OSF.IO/WKBXP). However, due to their size (~3 ​GB per file), the original EEG files are only available on request.
